# Variability in Clinical Phenotype in TARDBP Mutations: Amyotrophic Lateral Sclerosis Case Description and Literature Review

**DOI:** 10.3390/genes14112039

**Published:** 2023-11-04

**Authors:** Michele Lombardi, Lucia Corrado, Beatrice Piola, Cristoforo Comi, Roberto Cantello, Sandra D’Alfonso, Letizia Mazzini, Fabiola De Marchi

**Affiliations:** 1 ALS Center, Neurology Unit, Department of Translational Medicine, University of Piemonte Orientale, 28100 Novara, Italy; 20052002@studenti.uniupo.it (M.L.); roberto.cantello@med.uniupo.it (R.C.); letizia.mazzini@uniupo.it (L.M.); 2 Department of Health Sciences, University of Piemonte Orientale, 28100 Novara, Italy; lucia.corrado@med.uniupo.it (L.C.); beatrice.piola@uniupo.it (B.P.); sandra.dalfonso@med.uniupo.it (S.D.); 3 Neurology Unit, S. Andrea Hospital, Department of Translational Medicine, University of Piemonte Orientale, 13100 Vercelli, Italy; cristoforo.comi@med.uniupo.it

**Keywords:** mutation analysis, ALS, motor neuron diseases, familial ALS, TDP-43, TARDBP

## Abstract

Mutations in the 43 kDa transactive-response (TAR)-DNA-binding protein (*TARDBP*) are associated with 2–5% of familial Amyotrophic Lateral Sclerosis (ALS) cases. TAR DNA-Binding Protein 43 (TDP-43) is an RNA/DNA-binding protein involved in several cellular mechanisms (e.g., transcription, pre-mRNA processing, and splicing). Many ALS-linked *TARDBP* mutations have been described in the literature, but few phenotypic data on monogenic TARDBP-mutated ALS are available. In this paper, (1) we describe the clinical features of ALS patients carrying mutations in the *TARDBP* gene evaluated at the Tertiary ALS Center at Maggiore della Carità University Hospital, Novara, Italy, from 2010 to 2020 and (2) present the results of our review of the literature on this topic, analyzing data obtained for 267 patients and highlighting their main clinical and demographic features.

## 1. Introduction

Amyotrophic Lateral Sclerosis (ALS) is a rare neurodegenerative disorder with an undetermined cause characterized by the concomitant involvement of upper motor neurons (UMNs) in the cerebral cortex and lower motor neurons (LMNs) in the brainstem and the spinal cord. This disease has an aggressive course, with a median survival of about three to five years after its onset [1,2]. ALS can be classified as several variants based on the clinical onset: indeed, in addition to the classic form, with the involvement of UMNs and LMNs, others may have predominant UMN, LMN, or bulbar involvement or respiratory dysfunctions [3,4,5]. Only 5–10% of ALS cases are classified as familial (fALS), whereas the remaining 90% of cases are considered sporadic (sALS), as they appear to occur randomly throughout this community [6,7]. Most fALS forms are autosomal dominant, while others can be autosomal recessive or X-linked. In Europe, the predominant portion of fALS cases, consisting of about 40% of all cases, are caused by a mutation in *C9orf72* [8], while another 15–20% of fALS cases are caused by a mutation in *SOD1* (thus representing 1–2% of all sALS cases) [9]. Other well-known involved genes are *FUS*, which accounts for 5% of all fALS cases [10], and *TARDBP*, which accounts for 2–5% of cases. Altogether, over 30 genes have been implicated as being causative, increasing the risk of developing ALS, or accelerating the progress of the disease, but many of these account for only rare cases [11,12].

Pathologically, almost all ALS cases are characterized by aggregates of the transactive response DNA-binding protein (TDP-43) in the cytoplasm of neuronal and glial cells [13]. The protein TDP-43 is encoded by the gene *TARDBP*, which contains six exons (five codings) and is located on chromosome 1p36.22, with mutations identified in both sALS and fALS cases [14,15]. Mutations in *TARDBP* usually cause the degeneration and death of motor neurons in the brain and spinal cord due to the accumulation of such aggregates and the loss of nuclear TDP-43. The accumulation of these cytoplasmic bodies begins with the altered form of TDP-43, which is often ubiquitinated and forms the main component of such bodies [1]. 

Mutations in *TARDBP* in ALS patients account for around 2–5% of all ALS cases, placing it among the “screening genes” in patients with fALS. The *TARDBP* gene was initially identified as a 43 kDa transcription repressor that binds to the TAR-DNA element of HIV, and, because of its molecular weight of 43 kDa, it was named TDP-43 [16,17]. TDP-43 is a ubiquitously expressed DNA/RNA-binding protein [16] that contains two RNA recognition motifs, a nuclear localization sequence (NLS), a nuclear export signal [18], and a glycine-rich C-terminus that mediates protein–protein interactions [15,19]. This protein is involved in several cellular mechanisms, such as regulating RNA splicing and modulating pre-mRNA processing [14,20]. In addition to TDP-43 RNA, TDP-43 regulates the splicing and stability of a large number of other transcripts [21,22] and plays an important role in neuronal plasticity by regulating local protein synthesis in dendrites [23]. More than 30 mutations in the *TARDBP* gene have been found in both fALS cases, accounting for 4% of all fALS cases [1,14,15,24]. The most relevant studies showed the pathological hallmarks of TDP-43 mutation in ALS, which include nucleus-to-cytoplasmic mislocalization, the deposition of ubiquitinated and hyper-phosphorylated TDP-43 into inclusion bodies, protein truncation leading to the formation of toxic C-terminal TDP-43 fragments, and protein aggregation [25,26]. Therefore, such changes in the protein structure underlie the degeneration of motor neurons in ALS patients’ brains and spinal cords and that occurring in most neurodegenerative diseases [27]. According to reports from the literature, patients with *TARDBP* mutations have a broad clinical spectrum, including ALS, frontotemporal dementia (FTD), and parkinsonism, and variations in age of onset and disease progression. In this manuscript, we aim to describe the clinical variability in *TARDBP* mutations in a cohort of ALS patients evaluated at the tertiary ALS Centre at Maggiore della Carità Hospital, Novara, Italy, as well as present a review of the literature on the demographic and clinical features of ALS patients who are carriers of mutations in *TARDBP*. 

## 2. Presentation of Cases

### 2.1. Methodology

**Figure 1:**
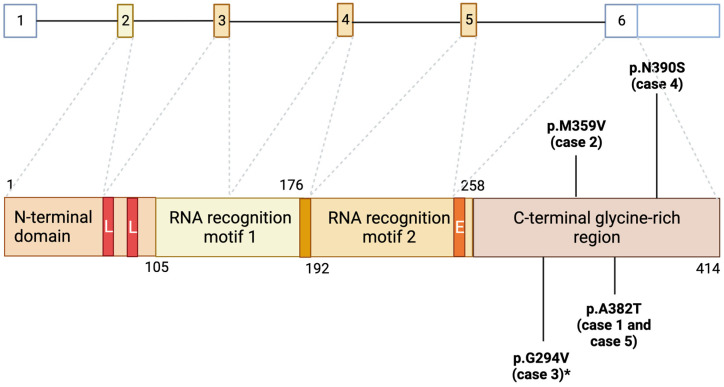
Structure of TDP-43 domain and the ALS-linked mutations reported in the described cases. Upper line: Coding regions (in yellow) and noncoding regions (in white) of *TARDBP* gene. Lower line: TDP-43 consists of an N-terminal domain, two tandem RNA recognition motifs (RRM1 and RRM2), and a C-terminal glycine-rich region. Most mutations in *TARDBP* are localized in the glycine-rich region. L: nuclear localization signal; E: nuclear export signal. Created at Biorender.com. * The mutation described in case 3 is the same mutation described by our group [28] for another patient with a homozygous status.

The patients described in this cohort analysis were all evaluated at the ALS Centre at Maggiore della Carità Hospital, Novara, Italy, and gave consent to be subjected to genetic analysis for research purposes. From 2010 to May 2023, we genotyped 592 patients. The gene analyses were performed at the Laboratory of Genetics, Department of Health Sciences, at the University of Piemonte Orientale, Italy. Six patients were carriers of *TARDBP* mutations (1%). One of these patients was previously described in the report by Corrado and colleagues, where they described the first ALS patient presenting the c.881 G>T p.G294V *TARDBP* mutation with a homozygous status [28]. In Figure 1, we show the structure of the TDP-43 domain and the position of the ALS-linked mutations observed in the described cases of our cohort.

### 2.2. Case No. 1

A patient who, at the age of 61, developed a slowly progressive muscle weakness in their left leg (distal involvement), with difficulty during walking, was initially observed by a general practitioner. After two years, the patient reported muscle weakness in their right leg as well, which was worse distally than proximally, leading to foot drop. He was otherwise well. He also reported a first-degree relative with MND. Upon neurological examination, we observed severe spasticity in the lower limbs, diffuse hyperreflexia, and left Babinski’s sign. However, no signs of muscle wasting, fasciculation, or upper limb or bulbar involvement were clinically evident. Due to a suspicion of possible MND, the patient underwent a brain and spinal cord MRI with DTI sequences, with a mild reduction in fractional anisotropy at the left posterior limb of the internal capsule; the EMG findings ruled out LMN involvement. Instead, the Motor Evoked Potentials (MEPs) of the lower limbs supported the diagnosis of MND due to a lack of central motor conduction and cortical potential. In addition, the brain FDG-PET did not show a significant degree of brain hypometabolism. Based on the clinical and instrumental findings, probable primary lateral sclerosis was diagnosed. The diagnosis was further confirmed genetically via the identification of a heterozygous missense variant in exon 6 of the *TARDBP* gene (c.G1144A, p.A382T, NM_007375). The patient is a slow progressor and was still able to walk after a 36-month follow-up. 

### 2.3. Case No. 2

A woman aged 55 presented to our department with a ten-month history of progressive lower-limb weakness. Neurological examination revealed fasciculations and hypotrophy, diffuse hyperreflexia, and spasticity, mainly in the lower limbs. No bulbar involvement was noted or indicated by the patient. Suspecting possible MND, the patient was subjected to MRI of the brain and spinal cord, which showed T2 hyperintensity of the corticospinal tract and hypodensity of the precentral cortex bilaterally. MEP and EMG findings also supported the diagnosis of MND involving both the UMN and LMN because of increased central motor conduction time and reduced cortical potential in the lower limbs and active denervation and chronic peripheral neurogenic degeneration in the cranial, cervical, and lumbar regions. Based on the clinical and instrumental findings, a diagnosis of prevalent UMN disease was made. The diagnosis was then genetically defined via the presence of a missense variant with a heterozygous status in exon 6 of the *TARDBP* gene (c.1075A>G. p.M539V, NM_007375).

### 2.4. Case No. 3

A man presented to the neurology department at the age of 64 years due to dysphonia and dysphagia. His family history did not indicate previous neurodegenerative diseases. On neurologic examination, we noted diffuse muscle weakness and hypotrophy, fasciculation, hyperreflexia, and spasticity of the upper and lower limbs. Neurophysiological signs of LMN and UMN were also present due to the lack of central motor conduction and cortical potential derived from the right leg and the reduction in cortical potential derived from the left leg, with mild signs of denervation of the lower limbs. MRI results for the brain were unremarkable. Based on the clinical and instrumental findings, a definite case of ALS with bulbar onset was diagnosed. The diagnosis was then confirmed genetically by identifying a heterozygous missense variant in exon 6 of the *TARDBP* gene (c.881 G>T p.G294V, NM_007375). He showed a 2-year history of rapidly worsening disease, with the occurrence of severe dysphagia and respiratory muscle weakness, until respiratory failure occurred. 

### 2.5. Case No. 4

A 72-year-old man presented to our department with a 20-year history of slowly progressive dysarthria and dysphagia that had developed beginning at age 51. After 7 years, the patient began to experience weakness and spasticity of the lower limbs to the point of developing a complete inability to walk. Neurologic examination did not reveal clinical signs of upper-limb involvement. No EMG signs of lower MN involvement were detected, nor was cognitive involvement found through neuropsychological tests. The brain MRI results were unremarkable; instead, upper- and lower-limb MEPs supported a diagnosis of MND based on the absence of central motor conduction and cortical potential. Based on the clinical and instrumental findings, a diagnosis of probable primary lateral sclerosis was made. The diagnosis was then defined genetically via the presence of a heterozygous missense variant in exon 6 of the *TARDBP* gene (c.1169A>G, p.N390S, NM_007375). 

### 2.6. Case No. 5

A 63-year-old-man presented to our department with a two-year history of slowly progressive dysarthria with occasional episodes of dysphagia, as reported by the patient. He also reported that a first-degree relative had died of MND. At this time, neurologic examination showed no significant muscle involvement other than minimal tongue hypotrophy and diffuse hyperreflexia. After four years, bulbar symptoms slowly worsened, associated with a paraparetic gait and cognitive involvement as assessed through neuropsychological testing. Suspecting possible MND, the patient was subjected to a brain and spinal cord MRI, the results of which were unremarkable. Instead, EMG findings supported a diagnosis of MND based on the presence of chronic peripheral neurogenic degeneration affecting the muscles in cranial, cervical, and lumbar districts. Based on the clinical and instrumental findings, a probable diagnosis of amyotrophic lateral sclerosis was made. The diagnosis was then genetically defined via detecting a heterozygous *TARDBP* mutation in exon 6 (c.G1144A, p.A382T, NM_007375). The patient died 50 months after diagnosis.

Table 1 summarizes the main clinical and demographic features of each described case. 

## 3. Literature Review

To review the phenotypes of *TARDBP* mutation carriers, two independent operators performed a PubMed search from 2008 (concerning initial descriptions of mutations in *TARDBP*) until May 2023. We identified 39 reports describing mutations considered pathogenetic for ALS. 

### 3.1. Statistical Analysis

Data are presented as the mean ± standard deviation (SD) or median ± interquartile range (IQR). All categorical variables are presented as numbers (percentages). For continuous variables, we used the Kolmogorov–Smirnov test to explore the normality of the distribution of the data. Due to the non-normality of the distribution of most variables, the Kruskal–Wallis and the Mann–Whitney U tests were used for continuous data comparison. Statistical significance was set at a *p*-value < 0.05. IBM SPSS software (version 25.0, IBM Corporation, Armonk, NY, USA) was used for the statistical analysis. 

### 3.2. Review of Clinical Phenotypes of TARDBP Mutations in Cases of ALS

In our review of the literature, which solely included people with ALS, we identified 50 different mutations derived from 39 articles or reviews, most of them from Italian or Chinese groups. Almost all the mutations were genetically defined as missense variants in heterozygous exon 6 of the *TARDBP* gene, with the exception of the detection of a truncation variant at the extreme C-terminus of the protein p.Y374X [29] and a variant in exon 2 [30].

Familial data were available for 267 patients (all individual cases are described in the Supplementary Materials), of whom 127 (47.6%) were diagnosed as familial cases, because there was at least one other described case in the same family, and 140 (52.4%) were diagnosed as sporadic.

Due to their lack of sufficient clinical data, four articles were excluded from the statistical data analysis, leaving available clinical data for 246 patients (92%). Of these, gender was available for 230 patients (93.5%). Of these, 138 (60%) were male and 92 (40%) were female. Age at onset was available for 231 patients (94%). The mean age at onset was 53.94 years (SD: 12.34), with no differences between fALS and sALS cases (*p*-value > 0.05). The region of onset was available for 218 patients (88.6%). Of these, 152 (70%) had a spinal onset, while only 66 (30%) had a bulbar onset. None had a respiratory onset. Among the most common phenotypes presented in spinal patients, 66 of 94 (70.2%) had a predominant UMN phenotype, whereas only 28 (29.8%) had a predominant LMN phenotype. Only one article described a concomitant presence of extrapyramidal symptoms belonging to the Parkinson’s spectrum [31]. Cognitive status was assessed only in a percentage of cases (N = 167, 67.9%); of these, 21 (12.6%) had cognitive involvement (not classified by Strong’s criteria; only three articles clearly investigated the presence of FTD criteria [31,32,33]). Disease duration was described for 191 patients (77.6%), with a wide variability between the various cases described. The median disease duration was 38.00 months (IQR: 21–78.5).

**Figure 2:**
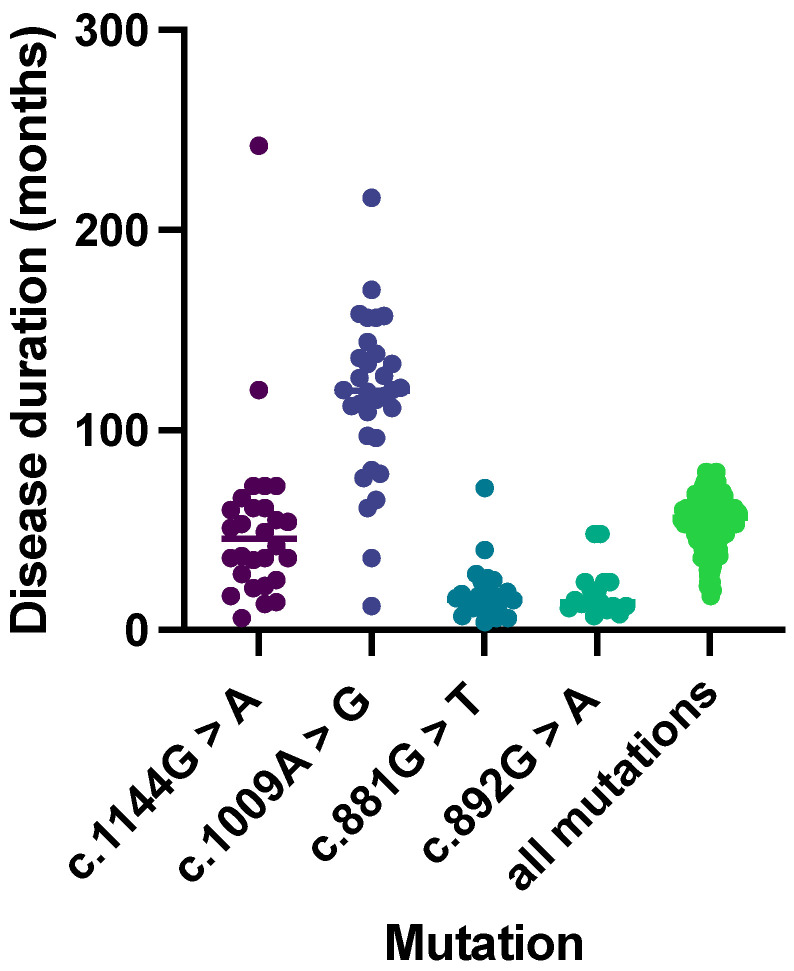
Disease duration (in months) of patients carrying different mutations in the *TARDBP* gene. Among the group of carriers, those with the c.1009A>G mutation had a statistically longer survival period compared to the others.

Among the heterogeneity of the mutations recorded, four of them accounted for 51% of all cases (N = 133). The most frequent ones are as follows: c.1009A>G (16.9%; N = 45), c.1144G>A (14.6%; N = 39), c.881G>T (10.9%; N = 29), and c.892G>A (8.6%; N = 23). So, we analyzed and compared patients grouped by mutation:

- c.1009A>G, p.M337V: Clinical data were available for 34 patients; of them, 31 (91%) were determined to be familial. Gender was available for 33 patients. Of these, 15 (45%) were male, and 18 (55%) were female. The mean age at onset was 50.33 years (SD: 9.04). Only 13 patients (38%) had a spinal onset, in contrast to the results previously shown for the whole group. Instead, the median disease duration was significantly (*p*-value < 0.001) higher than the rest of the group, amounting to 120 months (IQR: 96.75–136.50) (Figure 2). Cognitive involvement was found in only two patients.
- c.1144G>A, p.A382T: Only 10 (26%) cases were familial. A total of 28 (71.8%) were male, and 11 (28.2%) were female. The mean age at onset was 56.51 years (SD: 13.58). When taking the whole group into consideration, it was observed that cases with spinal onset predominated (N = 26; 66.7%). Disease duration was described for 36 patients (92.3%), with large variability between the different cases described (min: 6 months; max: 242 months). The median disease duration was 46 months (IQR: 27.25–61.00). Cognitive status was assessed in 24 patients: of these, 6 (25%) had cognitive involvement. These data are similar to those reported in a recent Italian study [34].
- c.881G>T, p.G294V: The number of familial cases (N = 13; 43.3%) was similar to that for sporadic cases (N = 17; 56.7%). Gender was available for 28 patients. Of these, 18 (62.1%) were male, and 11 (37.9%) were female. The mean age at onset was 61.79 years (SD: 7.84). There were no significant differences between bulbar (N = 15; 51.7%) and spinal (N = 14; 48.3%) onset. Disease duration was described for 27 patients (90%), with wide variability between the different cases described. The median disease duration was 15 months (IQR: 12.0–21.5), significantly (*p*-value < 0.001) lower than that in the other described cases. Cognitive impairment was found in only 2 of the 14 (46.7%) clearly studied patients.
- c.892G>A, p.G298S: Clinical data were available for 22 patients. Of them, 13 (59.1%) were identified as familial. Gender was available for 21 patients. Of these, 11 (52.4%) were male, and 10 (47.6%) were female. The mean age at onset was 51.29 (SD: 8.07). Only 13 patients (59.1%) presented with a pure spinal onset. Disease duration was described for 17 patients (77.3%). The median disease duration was 14 months (IQR: 12–24), significantly (*p*-value < 0.001) lower than the rest of the records. Cognitive status was evaluated in 21 (95.5%) patients, and none of them showed cognitive impairment.

No significant differences (*p*-value > 0.05) were detected in any of the mutations described above in terms of age at onset.

### 3.3. Neuroradiological Studies

Until now, there has been a lack of comprehensive and definite MRI descriptions for TARDBP-MND patients. Various patterns of atrophy have been reported, primarily aligning with an individual’s clinical presentation and cognitive impairment. In 2021, Agosta’s group [35] conducted a volumetric MRI analysis on a cohort of TARDBP patients (N = 10), revealing a significant absence of gray matter involvement in ALS-TARDBP patients, in contrast to rare instances where frontotemporal atrophy was observed only in FTD-TARDBP individuals [36,37]. Recently, the same group [38] described their imaging findings obtained for 11 patients using advanced MRI techniques, unveiling a distinctive parietal pattern and notable damage to both motor and extra-motor white matter regions in these mutated patients. Specifically, a whole-brain voxel-based morphometry analysis indicated substantial atrophy in the right lateral parietal cortex as well as reduced gray matter volumes in the left precuneus and right angular gyrus in the TARDBP subjects compared to healthy controls. Additionally, diffusion tensor imaging demonstrated decreased fractional anisotropy in the right corticospinal tract in TARDBP patients, along with higher axial diffusivity values in the left inferior longitudinal fasciculus, distinguishing them from the control group. 

FDG-PET scans have shown potential as a tool for detecting early changes in brain metabolism that correlate with clinical phenotype in genetic ALS patients. Only one study, conducted by Canosa and colleagues [39], explored metabolic changes in the brains of 14 patients with mutations in *TARDBP* (p.A382T) using FDG-PET. The authors identified relative hypometabolism in mutated patients, specifically in the right precentral and postcentral gyrus, superior and middle temporal gyrus, and insula, when compared to control ALS patients, suggesting a potential link with mild cognitive changes. 

### 3.4. Comparison with Other Monogenic Motor Neuron Diseases

It is known that more than 30 genes, most of which are passed down through autosomal dominant inheritance, have been identified as causing or contributing to the development of ALS. However, many of these genes are only responsible for rare cases [11,12]. Among familial cases, which account for 10–15% of ALS [7], the predominant portion, consisting of about 60% of all cases, is caused by a mutation in the *C9orf72* or the *SOD1* gene, accounting for 40% and 15–20% of all fALS cases, respectively [8,9]. In contrast, *TARDBP* accounts for only 2–5% of cases. Knowing the phenotypic expression of such mutations, which appear to differ slightly as already reported in the literature, could guide clinicians in targeting genetic investigations and determining the clinical evolution and prognoses of these forms to establish personalized treatment plans suitable for each patient.

Based on the data extrapolated from the literature and provided in more detail in the Supplementary Materials, the most prevalent TARDBP-mutated phenotype appears to be male, with a mean age at onset of 54 years and a median disease duration of 38 months. Clinically, the disease primarily presents with a purely spinal onset, often beginning in the upper limbs and progressing towards a classic form involving both upper and lower motor neurons. Extrapyramidal signs are extremely rare, and cognitive impairment affects a minimal portion of cases. 

When examining variations between different mutations, *SOD1* mutations appear to be slightly more prevalent in females. SOD1 patients tend to be younger than other ALS patients [40] and have a longer survival compared to patients without ALS-associated gene mutations (except for the mutations p.L39V, p.G42S, p.G73S, and p.D91N [41]). The typical clinical presentation involves the lower limbs and, like TARDBP cases, evolves toward a classical form of ALS. Cognitive impairment is very rare [42]. Extrapyramidal signs are described in only a few cases among the SOD1-mutated patients. 

C9orf72-mutated patients typically experience an earlier symptom onset with the other mutations discussed above and a significantly lower disease duration [43]. In terms of the site of symptom onset, bulbar onset is more common than spinal onset. Notably, in contrast to the previously mentioned mutations, *C9orf72* mutations are more frequently associated with FTD or cognitive impairment. Some studies have reported that up to 50% of the analyzed familial cases with *C9orf72* mutations exhibited FTD [44,45]. In sporadic ALS cases, the likelihood of FTD is lower, affecting only around 20–25% of patients. Additionally, the *C9orf72* mutation is also associated with other neurological conditions, such as Parkinson’s disease, progressive supranuclear palsy, ataxia, corticobasal syndrome, Huntington-disease-like syndrome, Creutzfeldt–Jakob disease, and Alzheimer’s disease [46]. *C9orf72* mutations appear to be extremely rare in Asia and the Middle East, in contrast to *TARDBP* and *SOD1* mutations, which are globally widespread, with slight variations between different mutations [47].

## 4. Conclusions

In summary, our first objective was to outline the clinical and demographic features of the ALS-TARDBP patients within our cohort. Additionally, the literature review revealed a diverse range of clinical phenotypes and variability among ALS patients with *TARDBP* mutations. Given the high prevalence of *TARDBP* mutations in patients with a predominant UMN phenotype and the recent advancements in precision medicine and genetic therapies, we advocate for the consideration of genetic testing as a routine part of the diagnostic work-up for all patients with MNDs, including those displaying clinical features consistent with primary lateral sclerosis or, more broadly, prominent UMN involvement. The evolving landscape of gene discovery has complicated and empowered the role of genetics in the care of ALS patients, necessitating genetic analyses and related counseling. We believe that these aspects are increasingly pivotal in managing both sporadic and familial ALS cases. Lastly, in the case of the rare *TARDBP* mutations, future research, incorporating a larger cohort of patients carrying these mutations and longitudinal data, will help to confirm our findings. 

## Figures and Tables

**Table 1:** The clinical phenotypes of patients diagnosed at Maggiore della Carità University Hospital are summarized in the Table. LMN: lower motor neuron; UMN: upper motor neuron. N/A: not applicable.

	CASE 1	CASE 2	CASE 3	CASE 4	CASE 5
**Age at onset (years)**	61	55	66	72	63
**Symptoms at onset**					
**bulbar involvement**	no	no	dysphonia, dysphagia	dysarthria	dysarthria
**upper-limb involvement**	no	no	no	no	no
**lower-limb involvement**	spasticity	weakness	no	no	no
**Diagnostic delay (year)**	6	4	2	21	2
**Progression**	slow	fast	fast	slow	fast
**Cognition**	none	none	N/A	none	mild
**EMG**	negative for LMN signs	positive for UMN signs	mild LMN signs	negative for LMN signs	positive for cranial, cervical, and lumbar signs
**PEMs**	no cortical potential in lower limbs	reduced cortical potential derived from lower limbs (right > left)	no cortical potential derived from right leg; reduced from left leg	reduced cortical potential in upper and lower limbs	N/A
**MRI**	mild FA reduction (left internal capsule)	pyramidal tracts hyperintensity	unremarkable	unremarkable	unremarkable
**FDG-PET**	no brain hypometabolism	N/A	N/A	N/A	N/A
**Mutation**	c.G1144A, p.A382T, NM_007375	c.1075A>G p.M359V, NM_007375	c.881 G>T p.G294V, NM_007375	c.1169A>G p.N390S, NM_007375	c.G1144A, p.A382T, NM_007375
**Familial cases**	1 (father); no clinical evidence in siblings and son	none	none	none	1 (brother); no clinical evidence in parents and son

## Data Availability

Datasets analyzed during the study can be obtained from the corresponding author upon reasonable request.

## Supplementary Materials

The following supporting information can be downloaded at https://www.mdpi.com/article/10.3390/genes14112039/s1. Table S1: Review of all cases published in the literature, described as single-subject. Refs. [48,49,50,51,52,53,54,55,56,57,58,59,60,61,62,63,64,65,66,67,68] are cited in the supplementary materials.
